# Right Wing Politics and Public Policy: The Need for a Broad Frame and Further Research Comment on "A Scoping Review of Populist Radical Right Parties’ Influence on Welfare Policy and its Implications for Population Health in Europe"

**DOI:** 10.34172/ijhpm.2020.137

**Published:** 2020-07-26

**Authors:** Ronald Labonté, Fran Baum

**Affiliations:** ^1^School of Epidemiology and Public Health, University of Ottawa, Ottawa, ON, Canada.; ^2^Southgate Institute of Health, Society and Equity, Flinders University of South Australia, Adelaide, SA, Australia.

**Keywords:** Politics of Health, Social Determinants, Health Equity

## Abstract

Our paper responds to a narrative review on the influence of populist radical right parties (PRRPs) on welfare policy and its implications for population health in Europe. Five aspects of their review are striking: *(i) *welfare chauvinism is higher in tax-funded healthcare systems; *(ii)* PRRPs in coalition with liberal or social democratic parties are able to shift welfare reform in a more chauvinistic direction; *(iii)* coalitions involving PRRPs can buffer somewhat the drift to welfare chauvinism, but not by much; *(iv)* the European Union (EU) and its healthcare policies has served somewhat as a check on PRRPs’ direct influence on healthcare welfare chauvinism; *(v)* PRRPs perform a balancing act between supporting their base and protecting elected power. We note that PRRPs are not confined to Europe and examine the example of Trump’s USA, arguing that the Republican Party he dominates now comes close to the authors’ definition of a PRRP. We applaud the authors’ scoping review for adding to the literature on political determinants of health but note the narrow frame on welfare policy could be usefully expanded to other areas of public policy. We examine three of such areas: the extent to which policy protects those who are different from mainstream society in terms of race, ethnicity, gender or sexuality; the debate between free trade and protectionism; and the rejection of climate change science by many PRRPs. Our analysis concludes that PRRPs promote agendas which are antithetical to eco-socially just population health, and conclude for a call for more research on the political determinants of health.

Rinaldi’s and Bekker’s paper shines an analytical light on the welfare policy consequences of the rise of ‘populist radical right’ parties (PRRPs).^1^ A scoping review, their study attempts to assess the extent to which PRRs engage in ‘welfare chauvinism,’ a term used to describe how some groups are favoured (the ‘in-group’) and others excluded (the ‘out-group’) from welfare entitlements. The term originated in the 1990s in studies of radical right parties in Western Europe^2^ and continues to generate considerable attention from (primarily European) political scientists. Welfare chauvinism is similar to earlier notions of ‘deserving’ and ‘undeserving’ poor, in which assistance is withheld from the ‘undeserving’ able-bodied who refuse to work however menial the labour or impoverishing the wages. Welfare minimalism emphasizing labour attachment persists across a range of political parties, notable in the near global reach of neoliberal austerity measures post-2008,^3^ only recently suspended in response to the near global reach of the severe acute respiratory syndrome coronavirus 2 (SARS-CoV-2) pandemic. But welfare chauvinism departs from earlier demarcations of deservedness based poverty or employment. Instead, it invokes the increasingly racialized (xenophobic) identity politics in separating the ‘in’ from the ‘out’: the ‘native-born’ from the ‘immigrant,’ the ‘pure people’ from the ‘corrupt elite,’ and the ‘white’ (predominantly male) from everyone else. It also favours authoritarian rule provided, of course, that it is supplied by one of the ‘in.’ In Europe this construction of acceptability based on race raises the unhappy spectre of fascism as it was expressed in the 1930s and 1940s when German public policy evolved designed to exterminate groups including Jewish people, Roma, and those with intellectual or physical disability. Hence the importance of this paper in unpicking the current rise in PRR and their impact on welfare policies.

 Rinaldi’s and Bekker’s analysis focuses on Europe but this rise of fascist leaning parties in not only seen there. Hence their important and timely review of the extant empirical evidence of PRRP influence on European welfare policy has much broader implications. Although health (primarily healthcare) policy is the focus of a few of their cited studies, the authors use the broader sweep of welfare policy reforms as a proxy for population health and health equity. This is a reasonable assertion, given that welfare policies play an important role in determining health outcomes. Their primary rationale, however, is that there is a “lack of literature about the direct relationship between PRR [populist radical right] parties and health” (p. 2). This acknowledgement points to a limitation of scoping reviews: they can only assess or analyze the evidence gathered by others’ research and thus are confined to the questions, methods, and findings provided by such studies.

 That limitation notwithstanding, some of their findings are striking and bear further commentary:

1. Welfare chauvinism is higher in tax-funded healthcare systems. This finding is consistent with what one of us recently described as ‘primordial neoliberalism,’ where the individualism and responsibilization associated with neoliberal austerity coalesces with nativist politics and an (apparent) retreat from globalization’s permeable economic borders.^4^
2. PRRs in coalition with liberal or social democratic parties are able to shift welfare reform in a more chauvinistic direction, something widely seen beyond as well as within the European Union (EU) orbit. This finding, however, begs the empirical question: how much of a shift, and at what level of PRRP governing power within a coalition?
3. Coalitions involving PRRs can buffer somewhat the drift to welfare chauvinism, but not by terribly much. This may be an artefact of whether the dominant coalition party is conservative (the favoured allies of PRRPs) or social democratic. The review is fairly silent on this point, although it does refer to social democrats defeating conservative/PRRP coalitions partly by taking on some of their nativist rhetoric.
4. The EU and its healthcare policies (albeit resented by many EU member nations) has served somewhat as a check on PRRP direct influence on healthcare welfare chauvinism. This puts the UK Brexit in a new light to the extent it incentivizes more PRRs campaigning on an anti-EU platform (pretty much a mandatory PRRP policy plank). The UK Brexit campaign stressed the benefits for the National Health Service (NHS) of leaving the EU but managed to dodge the fact that many of the workers who staff the NHS are migrants.^5^
5. There is, however, a balancing act between supporting the base, and protecting elected power. The review proffers the example of PRRP support for conservative party coalition polities that target the ‘undeserving’ (the residual liberal/neoliberal dichotomy) but less so when these reduce provisions (such as pensions, healthcare, and labour market reforms). that would favour the PRRPs’ base. Social democratic parties, in turn, become more critical of migration policies to regain, or to avoid losing, votes to PRRPs.

Related to this last point, and one of the important insights raised in Rinaldi’s and Bekker’s review, is that of the political tension PRRPs face in balancing vote-seeking behaviours (appeals to their base) and office-seeking behaviours (avoiding blame for any welfare retrenchments coalition governments introduce that might negatively affect their base). The literature they cite is somewhat ambivalent on the success PRRPs have in juggling their need for votes and their necessity for governing compromise, although drawing attention to that is, in itself, is a valuable contribution.

Ironically, the best and least ambiguous example of trying to achieve both ends comes from a country and a political party the authors exclude in their boundary-setting PRRP definition: the United States and the Republican Party. Historically it may be correct to argue, as the authors do, that the policy platforms of the Republican Party are not strictly PRR, although in recent years (pre-dating the Trump administration) the Party’s swing to more extreme fiscally conservative positions bring it closer to a ‘radical right’ label. Even before Trump’s election the Republican Party increasingly belonged to its radical fiscal *and* socially conservative Tea Party base, often fomenting a racialized welfare chauvinism in rhetoric, if not also in policy. Coronavirus disease 2019 (COVID-19) has further revealed America’s PRR ugly face, manifest, for example, in vote-seeking trillion dollar pandemic bailouts for corporations (including tax breaks) and a one-time US$1200 cheque to citizens earning less than US$99000 annually, bearing Trump’s own signature. Although there is now talk of a second one-off cheque,^6^ most assessments contend that America’s corporate sector and uber-elite will benefit the most, exacerbating an already numbing history of income (and racialized) inequalities.^7-9^ At the same time the world continues to watch with tiring incredulity the multiple office-seeking efforts Trump has made to blame everyone or anything but himself or his administration for the US leading the world’s COVID-19 case and death count, while claiming the United States (personified in himself) has done best in managing the pandemic. Whether or not the Trump administration’s maneuverings around its nativist (and evangelical) base and its desire to continue governing (constrained only by the US Congress or Senate) is a question only the November US elections can answer. But the dilemma this poses for PRRPs offers some potential leveraging points for those opposed to PRRPs’ policy platforms. That, in itself, in an important contribution the paper makes.

 Immigrants and the broader tilt to xenophobic authoritarianism are central tenets in Rinaldi’s and Bekker’s review.

PRRPs, of course, are not the only political entities to restrict migrant rights or entitlements, a trend most commonly but not exclusively associated with conservative parties. In Australia the rise of a PPR party One Nation holding explicit racist views on migrants and Indigenous peoples was outflanked by the conservative Liberal Party then led by Prime Minister John Howard.^10^ This move undermined the previous bi-partisan policies which were benign on race and set the stage for off-shore detention camps for asylum seekers and the current refusal to provide welfare and publicly funded health services to asylum seeking refugees.^11^ Canada faced similar retrenchment of health coverage for asylum seekers under a federal conservative government, but opposition by public health professionals and a court decision reversed the budget cuts and exclusions.^12^ Right-wing columnists, however, continue to rail against ‘illegal migrants’ as ‘bogus asylum seekers.’^13^

PRRPs are more extreme with their anti-migrant policies, but even there, as Rinaldi and Bekker point out, they might hide their nativist biases by attacking welfare policies that indirectly affect migrants, rather than directly removing migrant-specific entitlements rights. Given the temporality of many of the studies in their review, there is reason to suspect that direct attacks on migrant policies might become more explicit. The advent of COVID-19 appears to have seen this happen in many countries,^14^ such that concern is being expressed that stigma against migrants and refugees is increasing. At the very least, there is growing evidence that the current pandemic has been used by some authoritarian regimes to strengthen their nativist policies (eg, Modi’s Hindu nationalism in India)^15^ or otherwise triggered increased racism in Europe targeting Asians, Jews, Muslims, Roma, and migrants as causes of COVID-19.^16,17^ Whether the renewed and globalizing anti-racism movement triggered by police violence against blacks, Indigenous, and ethnic minorities will damper this recent rise is another question central to debates about what a post-COVID world (and its political economies) should look like.

Since the advent of PPRPs it has been evident that more moderate conservative parties have tried to reduce the electoral losses to the PPRPs they might have faced by shifting to the right. In Australia the Liberal party moved to the right and allowed the PPRP One Nation to be openly critical of policies which they saw as advantaging Indigenous peoples without contradicting or challenging the divisive views.^18^ In the United Kingdom a similar move to the right partly in response to populist parties like UKIP (the pro-Brexit party) the British Conservative Party has also moved the right and become more populist. This move is seen in the rise of Boris Johnson himself a populist leader.^19^

One frustration with reading the paper was its narrow frame on welfare policies. While this is understandable within the confines of a single paper, it led us to consider other policy areas where PRR ideology would have an impact detrimental to health. For instance, PPRPs also reject much of the identity politics that have characterised human rights demands in many high-income countries in the past few decades. Identity politics includes recognition of a spectrum of sexualities, the importance of gender, embrace of differing ethnicities, and often recasting of colonial histories. An example is the ways in which Indigenous peoples in colonised countries have engaged in demands for decolonisation. These demands have clear implications for health.^20,21^ PRRPs in countries such as Canada, Australia, and Brazil position Indigenous peoples as “the other” and criticise any special measures designed to overcome their disadvantage.

Another area in which PRR parties appeal to the nativism identified by Rinaldi and Bekker is that of trade, where their position is often hostile to global trade agreements and in favour of economic protectionism. This poses a dilemma for public health advocates, since current trade and investment rules have been critiqued for their failure to protect public health regulatory and policy flexibilities posing considerable health risk. Moreover, these rules have disproportionately benefited global elites, partly by outsourcing much goods and services production from high-income to low- and middle-income countries.^22^ But there is an important distinction to be made between calling for reform of trade rules in the name of health equity and environmental sustainability, and in advocating economic protectionism on an assumption that closed borders will create new employment for those whose livelihoods were weakened or displaced by a global market integration disproportionately benefitting global elites.

A further policy area which has considerable implications for health is that of climate change policies and orientations towards decarbonising economies. Evidence suggests that right wing populist parties are climate sceptics^23^ and they attack what they see as hysteria over rising global temperatures. In much the same vein science skepticism has been seen in response to COVID-19. Both Boris Johnson and Donald Trump resisted acting on the advice of public health experts and only begrudgingly took some of the recommended measures when infections and deaths rose alarmingly.^24^ Each of these policy areas appears to represent another fruitful area of research in the emerging field of political determinants of health.

Aside from our interest in expanding analyses of PRRPs beyond welfare regimes, we acknowledge the several specific contributions Rinaldi and Bekker offer in their review, and its general contribution to the growing literature on the political determinants of health.^25^ Policies in all sectors have an impact on health and so are open to political influence. This was recognised by the Commission on the Social Determinants of Health (2008) which noted the importance of the distal determinants of health “the distribution of money, power and resources at global, national and local levels” which play an invisible but powerful role in determining how health is distributed. Other examples of research which has looked at political determinants of health include Lencucha and Thow’s^26^ consideration of how the institutionalisation of neo-liberalism has seen mechanisms enshrined which create structural barriers preventing governments from taking meaningful action to reduce the supply of unhealthy commodities; and Baum’s^27^ consideration of health governance with a chapter on its political dimensions. More political analysis of health issues is important because so often public health advocates call for great political will but rarely is effort expended on how this might be gained. Where this is done insights useful for opposition to those political determinants that are exclusionary, disequalizing, and unhealthy can be gained.^28^

 We urge public health researchers to pay more attention to the political determinants of health (both health affirmative and health destroying), and applaud Rinaldi and Bekker for opening up research on PRRPs whose agenda most often appears to be antithetical to eco-socially just population health.

## Ethical issues

 Not applicable.

## Competing interests

 Authors declare that they have no competing interests.

## Authors’ contributions

 Both authors equally contributed to the article.

## Authors’ affiliations

^1^School of Epidemiology and Public Health, University of Ottawa, Ottawa, ON, Canada. ^2^Southgate Institute of Health, Society and Equity, Flinders University of South Australia, Adelaide, SA, Australia.
